# Editorial: OMICS-based approaches in sports research volume II

**DOI:** 10.3389/fmolb.2022.1104142

**Published:** 2022-11-29

**Authors:** Mohamed A. Elrayess, Francesco Botrè, Amelia Palermo

**Affiliations:** ^1^ Biomedical Research Center, Qatar University, Doha, Qatar; ^2^ College of Pharmacy, QU Health, Qatar University, Doha, Qatar; ^3^ Laboratorio Antidoping, Federazione Medico Sportiva Italiana, Rome, Italy; ^4^ REDs-Research and Expertise in AntiDoping Sciences, Institute of Sport Sciences, University of Lausanne, Lausanne, Switzerland; ^5^ Department of Molecular and Medical Pharmacology, David Geffen School of Medicine, UCLA, Los Angeles, CA, United States

**Keywords:** sports and exercise medicine, omics, exercise, athletes, in vitro and in vivo models

OMICS approaches, including genomics, epigenomics, transcriptomics, proteomics, and metabolomics, continue to provide invaluable tools for better understanding of the molecular mechanisms underlying various physiological and pathological functions in health and disease. The rapid advancement of these tools and emergence of new ones is progressively filling the gap in our understanding of the complex networks that determine the structure, function, and dynamics of organisms. These advancements have greatly empowered discoveries of novel diagnostic and prognostic biomarkers as well as therapeutic targets, and provided a better guidance to precision and personalized medicine.

The utilization of OMICS approaches in sport science and medicine is becoming increasingly popular as the number of peer-reviewed original research articles has steadily increased over the last few years. Similar to complex phenotypes, athletic health and performance are greatly influenced by complex interactions between genetic predisposition and environmental factors, including diet, use of supplements and drugs, and type and intensity of training regiments. Indeed, the utilization of multi-OMICS tools in athletes has significantly informed training and predisposition to injury, identified novel biomarkers for endurance and power, and aided the detection of novel forms of doping substances. The combined utilization of OMCIS tools in various *in vitro* and *in vivo* models has greatly helped in determining the molecular mechanisms underlying the response to various stimuli relevant to sport science and medicine.

Following a successful volume I of OMCIS-based approaches in Sports Research, we extended our invitation for submissions targeting the following concepts 1) integrated omics in sport physiology and pathophysiology, 2) predictive biomarkers associated with improved athletic performance and on sport related risks on athlete’ heath and injuries, 3) investigation of hormones and growth factors for improved anti-doping tests, 4) identification of novel biomarkers for the detection of sport doping, 5) novel statistical/modelling approaches for omics data integration, interpretation and doping detection. The emerging publications in volume II have highlighted the importance of utilizing *in vitro* and *in vivo* models in sports OMICS to investigate and understand the effect of different factors on muscle function, energy homeostasis, immunomodulation, insulin signaling and ergogenicity.

Among the published articles in volume II, Gany et al. used untargeted metabolomics to study the effect of Tocotrienol supplementation on swimming time in aging rats and the underlying metabolic pathways. The study revealed that Tocotrienol caused a significant increase in swimming time in young rats. Aging was associated with upregulation of lipid metabolism and down regulation of energy and amino acid metabolism. However, Tocotrienol-supplemented rats exhibited upregulation of energy and amino acid metabolism in both adult and old rats, suggesting a favorable impact on the regeneration and renewal of skeletal muscle in ageing rats.

Another study by Belhaj et al. used untargeted metabolomics to investigate the metabolic pathways associated with disrupted AMPK-glycogen binding in transgenic mice in response to exercise. AMPK double Knock-In mice exhibited reduced maximal running speed and increased body mass and adiposity. Differences in plasma metabolic profiling were observed between resting and exercising transgenic mice and between wild type and transgenic mice at rest, while metabolite profiles of both genotypes converged following exercise. Differences in metabolite profiles were attributed to exercise-associated elevations in acylcarnitines and steroid hormones and reductions in amino acids and derivatives following exercise.

A third study by Li et al. investigated the effect of swimming on cancer induced muscle wasting in CT-26 cells-bearing mice. The study revealed that swimming attenuated tumor growth and muscle wasting, marked by elevated levels of various metabolites associated with anti-inflammatory and anti-apoptosis in quadriceps muscles.

A fourth study by Keller et al. used untargeted metabolomics to study the effect of nitrate exposure on hepatic amino acid and nutrient sensing pathways prior to exercise in zebrafish. The study revealed a greater abundance of metabolites involved in endogenous nitric oxide and amino acid metabolism in nitrate-treated livers at rest. Nitrate treatment also elevated the expression of genes involved in nutrient sensing, protein synthesis and purine metabolism, but reduced the expression of genes involved in mitochondrial fat oxidation. Data concluded that sub-chronic nitrate may improve exercise performance through increasing the bioavailability of nitric oxide, arginine sparing, and modulating hepatic gluconeogenesis and glycolytic capacity in the liver.

Finally, Alheidous et al. used *in vitro* models of preadipocytes and skeletal muscle cells to study the effect of sera from elite athletes who belong to different sport disciplines on cytokine secretion and insulin signaling in exercise-relevant tissues (adipose tissue and muscle). Sera from low power/low endurance as well as high power elite athletes induced TNF-α secretion in skeletal muscle cells, while sera from high endurance elite athletes reduced IL-6 secretion compared to non-athlete controls. All elite athlete sera groups caused decreased insulin sensitivity in preadipocytes, whereas in skeletal muscle cells, only sera from high endurance athletes reduced insulin signaling, while sera from low power/low endurance as well as high power elite athletes caused increased insulin sensitivity.

The emerging publications in volume II provide further evidence of the utility of applying OMICS approaches in relevant vitro and *in vivo* models to investigate the underlying metabolic pathways associated with various stimuli relevant to exercise physiology and pathophysiology.

